# Exploring the Factors Influencing Coronary Heart Disease Prevalence in the US Population: A Retrospective Observational Study

**DOI:** 10.7759/cureus.62741

**Published:** 2024-06-20

**Authors:** Mahima Kuruvila, Kavya Maddineni, Srija Reddy Koppula, Bianca Patel, Tanya Ratnani, Anumula Spandhana Reddy, Keethanshan Markandu

**Affiliations:** 1 Internal Medicine, Caribbean Medical University School of Medicine, Chicago, USA; 2 Public Health, Kent State University, Ohio, USA; 3 Cardiology, Kakatiya Medical College, Warangal, IND; 4 Pediatrics, Richmond University Medical Center, New York, USA; 5 Internal Medicine, Government Medical College, Bilaspur, IND; 6 Cardiology, Kamineni Academy of Medical Sciences and Research Centre, Hyderabad, IND; 7 Cardiology, Pennsylvania State University, Penn State College of Medicine, Penn, USA

**Keywords:** healthcare access, health status, socioeconomic factors, disparities, brfss, coronary heart disease

## Abstract

Introduction: Coronary heart disease (CHD) remains a significant global health concern and is characterized by inadequate blood supply to the myocardium due to the accumulation of plaque in the coronary arteries. Despite therapeutic advancements, prevalence disparities persist across various segments of the U.S. population, posing a significant challenge to healthcare systems. This study aims to find the prevalence disparities of CHD using Behavioral Risk Factor Surveillance System (BRFSS) data.

Methodology: A retrospective observational study was done using the 2022 BRFSS dataset on January 17, 2024. The study examined the presence of CHD as the dependent variable and investigated various independent variables. Descriptive and logistic regression analyses were conducted using the BRFSS Web Enabled Analysis Tool (Centers for Disease Control and Prevention, Atlanta, GA). Data management and storage utilized Microsoft Excel, and graphical analysis employed GraphPad Prism, version 9.4.1 (GraphPad Software, Inc., San Diego, CA).

Results: In demographics, respondents aged 65+ had higher CHD odds, while females exhibited lower risk than males. Hispanics had the lowest odds of CHD among all races. Socioeconomically, inability to work and retirees had higher CHD odds, as did income below $20,000 but ≥$15,000. Poor physical health increased CHD odds, as did having multiple healthcare providers. Medicare users had the highest CHD odds among insurance options.

Conclusions: Significant disparities in CHD prevalence were seen across demographic, socioeconomic, health status, and healthcare access dimensions in the United States, emphasizing the urgent need for targeted interventions to address these disparities and improve overall public health outcomes.

## Introduction

Coronary heart disease (CHD) represents a significant global health concern, constituting 2.2% of the overall disease burden and 32.7% of cardiovascular diseases [1]. CHD involves inadequate blood supply to the heart due to plaque buildup and is associated with comorbidities like high cholesterol, diabetes, smoking, obesity, and sedentary lifestyles [2]. While treatment modalities have advanced, health disparities persist, particularly affecting African American and Hispanic populations [3].

A Behavioral Risk Factor Surveillance System (BRFSS) survey highlighted disparities in CHD management, necessitating additional policy interventions for education and improved healthcare access, especially for disadvantaged groups [4]. CHD management has advanced, incorporating interventions like percutaneous revascularization and lifestyle changes such as blood pressure control and exercise, improving outcomes for individuals with CHD [5]. In another BRFSS survey, CHD patients reported their health journey and access to care. Disparities were noted among different demographics, impacting treatment access and outcomes; thus, even though medical management is improving there is an additional need to improve the humanistic burden of disease [6,7].

Objectives

The present study aimed to explore how CHD prevalence varies across demographics, socioeconomic factors, and health conditions using BRFSS data.

Additionally, the study also aimed to evaluate the association between CHD and health status/healthcare access and identify any potential disparities in CHD management among racial and socioeconomic groups.

## Materials and methods

Study design and setting

A retrospective observational study was conducted using the BRFSS database, accessed on January 17, 2024 [8,9]. All data from BRFSS were public and did not reveal any personal information, thus exempting the study from ethics committee approval [10].

Study tool and data collection

The BRFSS is a comprehensive health-related telephone survey system that collects data from U.S. residents about their health-related risk behaviors, chronic health conditions, and use of preventive services. The BRFSS Web-Enabled Analysis Tool (WEAT) database is an accessible online platform that allows users to analyze BRFSS data efficiently. The present study examined 438,693 respondents from the 2021 dataset, specifically those who answered *Yes* to the question regarding a history of angina or CHD.

The dependent variable in our study was the existence of chronic health care conditions (angina or CHD) molded by an affirmative answer to the question "Ever told you had angina or CHD" (CVDCRHD4). Independent variables in our study included demographic characteristics, socioeconomic characteristics, health status, and healthcare access. Demographic variables were age (calculated variable for a six-level age category), gender (SEX1) coded by (male, female), and race (variable for an eight-level race). Socioeconomic variables were education level (EDUCA) graded by six levels, ranging from never attended school to college graduate; employment status (EMPLOY1) was graded by eight levels, ranging from employed for wages to unable to work; annual household income (INCOME 3) graded by 11 levels, ranging from <$10,000 to ≥$200,000.

A calculated variable determined mental health status for three levels of not good mental health status (_MENT14D). Physical health status was determined by a calculated variable for three levels of not-good physical health status (_PHYS14D). Healthcare access was determined by responses to the following three questions: (1)* Do you have one person or group of doctors that you think of as your healthcare providers* (PERSDOC3) (responses included yes only one, more than one, no). (2) *Current primary source of your health insurance* (PRIMINSR) (11 responses ranging from a plan purchased through employer/union to no coverage of any type). (3) *In the past 12 months, I needed to see a doctor but could not afford* (MEDCOST1) (responses were yes, no).

Data and statistical analyses 

Descriptive and logistic regression analyses were also performed using the WEAT in the BRFSS. Logistic regression analysis was used to assess the independent variables studied. The analysis utilized t-tests, odds ratios (ORs), and 95% confidence intervals (CIs), with a *P*-value < 0.05 considered significant. Data were stored in Microsoft Excel, and graphical analysis was performed in GraphPad Prism, version 9.4.1 (GraphPad Software, Inc., San Diego, CA).

## Results

The study initially involved 438,693 respondents surveyed in the year 2021. Among them, 22,891 respondents were confirmed to have a history of angina or CHD based on their affirmative response to the relevant survey question (Table 1).

**Table 1:** Demographic and socioeconomic characteristics of the study population. GED, general educational development

Variable	*n* (%)
Demographic (*n* = 22,891)	
Age (years)	
18-24	84 (1.2)
25 to 34	230 (2.3)
35-44	524 (3.9)
45-54	1,612 (10.9)
55-64	4,271 (22.7)
65 or older	16,170 (59)
Gender	
Male	13,271 (58.1)
Female	9,620 (41.9)
Race	
White, non-Hispanic	19,011 (75.4)
Black, non-Hispanic	1,439 (9.5)
Hispanic	1,027 (8.9)
American Indian/Alaskan Native, non-Hispanic	383 (1.1)
Asian, non-Hispanic	211 (2.8)
Native-Hawaiian/Other Pacific Islander, non-Hispanic	80 (0.2)
Other race, non-Hispanic	282 (0.9)
Multiracial, non-Hispanic	458 (1.3)
Socioeconomic parameters	
Education level (*n* = 22,799)	
Never attended school or only kindergarten	27 (0.3)
Grades 1-8 (elementary)	575 (5.1)
Grades 9-11 (some high school)	1,236 (10)
Grade 12 or GED (high school graduate)	6,622 (29.9)
College 1-3 years (some college or technical)	6,689 (32.5)
College 4 years or more (college graduate)	7,650 (22.2)
Employment status (*n* = 22,593)	
Employed for wages	3,335 (18.2)
Self-employed	1,321 (6.1)
Out of work for 1 year or more	478 (2.6)
Out of work for less than 1 year	237 (1.3)
A homemaker	574 (2.9)
A student	46 (0.3)
Retired	13,537 (50.9)
Unable to work	3,065 (17.6)
Annual household income (*n* = 18,156)	
Income < $10,000	770 (5.5)
$10,000 <= Income < $15,000	1,105 (6.3)
$15,000 <= Income < $20,000	1,276 (7.2)
$20,000 <= Income < $25,000	1,703 (9.2)
$25,000 <= Income < $35,000	2,919 (14.8)
$35,000 <= Income < $50,000	2,778 (14)
$50,000 <= Income < $75,000	2,988 (15.3)
$75,000 <= Income < $100,000	2,007 (11.2)
$100,000 <= Income < $150,000	1,551 (9)
$150,000 <= Income < $200,000	527 (3.8)
Income >= $200,000	532 (3.7)

Demographic characteristics

**Figure 1:**
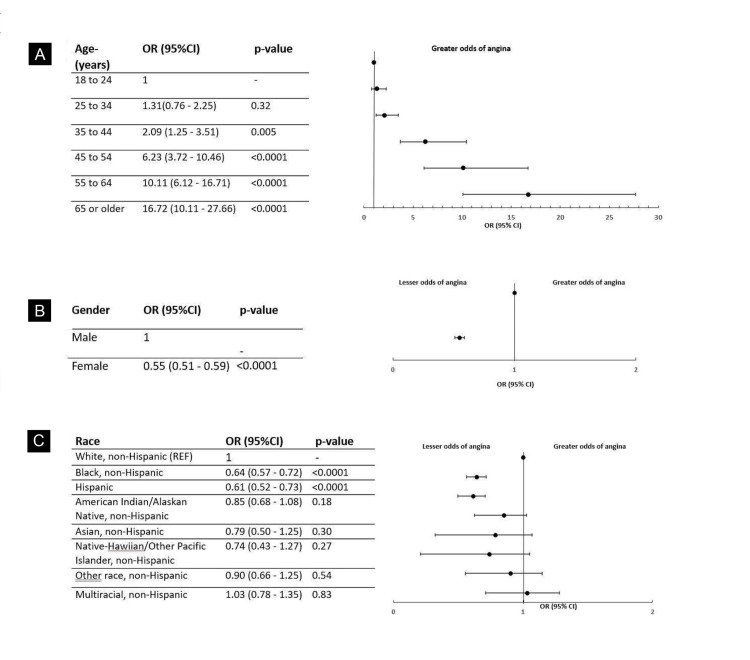
Odds of CHD stratified by demographic and socioeconomic characteristics (A) age, (B) gender, and (C) race. CHD, coronary heart disease; CI, confidence interval; OR, odds ratio

Among the study respondents with a history of CHD, 16,170 (59%) were 65 years or older. Compared to the reference age group of 18 to 24 years, the odds of having CHD in the age group 35 to 44 years was (OR, 2.09; 95% CI, 1.25-3.51; *P*-value = 0.005), while the odds were significantly high in the respondents aged 65 years or older (OR, 16.72; 95% CI, 10.11-27.66; *P*-value < 0.0001) (Figure 1A). A total of 13,271 (58.10%) respondents were males. Females exhibited significantly lower odds of having CHD compared to males (OR = 0.55; 95% CI, 0.51-0.59; *P*-value* *< 0.0001) (Figure 1B). Black, non-Hispanic respondents had significantly lower odds (OR, 0.64; 95% CI, 0.57-0.72; *P*-value < 0.0001) of having a history of CHD compared to the reference group, White, non-Hispanic. A similar trend was observed among Hispanic individuals (OR = 0.61; 95% CI, 0.52-0.73; *P *< 0.0001) (Figure 1C).

Socioeconomic characteristics

**Figure 2:**
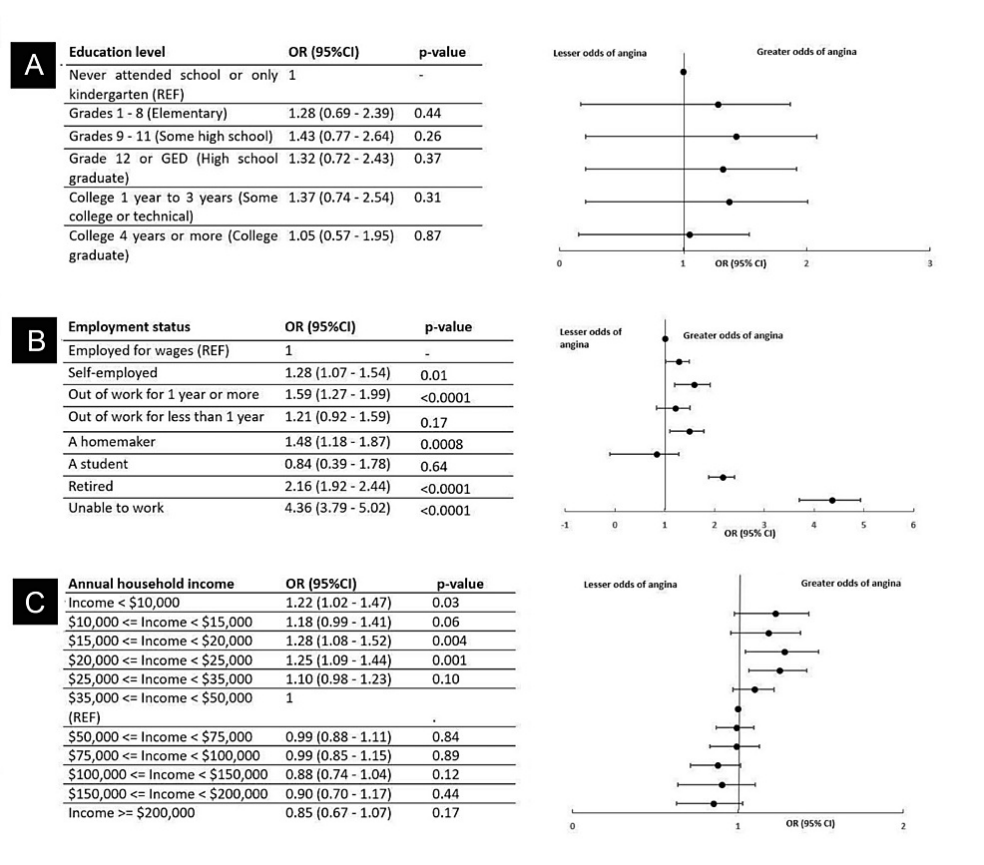
Odds of CHD stratified by demographic and socioeconomic characteristics: (A) education level, (B) employment status, and (C) annual household income. CHD, coronary heart disease; CI, confidence interval; OR, odds ratio

Among the sample of 22,799 respondents with CHD, no significant relationship between education level and history of CHD has been identified (*P* > 0.05) (Figure 2A). Considering the employment status among 22,593 respondents, those who were unable to work had significantly higher odds of CHD (OR, 4.36; 95% CI, 3.79-5.02; *P* < 0.0001), followed by retirees exhibiting a similar trend (OR, 2.16; 95% CI, 1.92-2.44; *P* < 0.00001 (Figure 2B). Moreover, 1,276 (7.20%) of respondents among the sample of 18,156 respondents with an annual household income lower than $20,000 but greater than or equal to 15,000 had significantly higher odds of having CHD (OR, 1.28; 95% CI, 1.08-1.52; *P *= 0.004), while there was no significant relationship between income and history of CHD among respondents with income over $50,000 compared to the reference group (Figure 2C).

Health status

**Figure 3:**
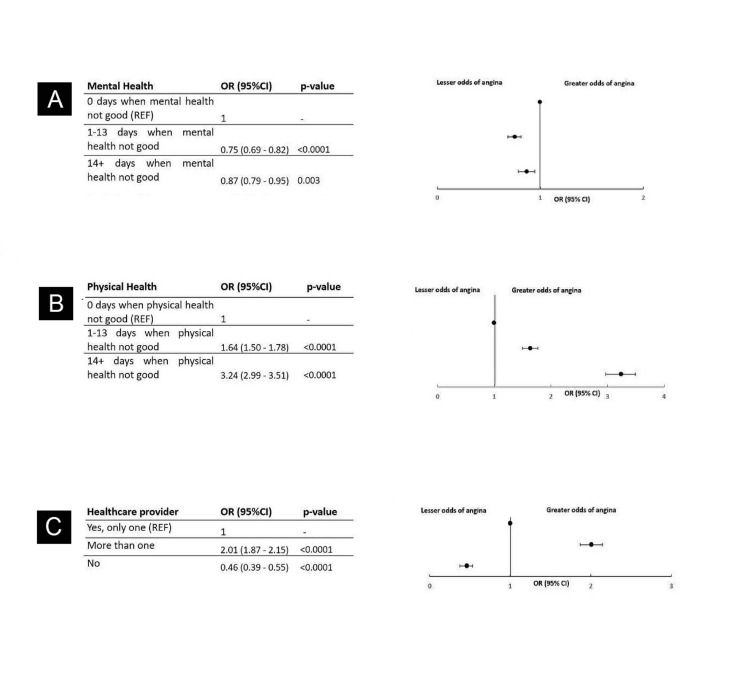
Odds of CHD stratified by health status and healthcare access: (A) mental health, (B) physical health, and (C) healthcare provider. CHD, coronary heart disease

Of the 22,353 respondents with CHD involved in the study, individuals experiencing 14 or more days of poor mental health had higher odds (OR, 1.33; 95% CI, 1.22-1.44; *P *< 0.0001) of CHD compared to those with 0 days of poor mental health (reference group) (Figure 3A; Table 2). A total of 6,747 (31.70%) of the 22,033 respondents who experienced 14 or more days of poor physical health faced significantly higher odds of CHD (OR, 4.84; 95% CI, 4.50-5.20; *P *< 0.0001) compared to the reference group of who reported 0 days of poor physical health (Figure 3B). Additionally, 10,969 (48.7%) of the total 22,753 respondents, with more than one healthcare providers, had significantly higher odds of CHD (OR, 2.38; 95% CI, 2.24-2.54; *P *< 0.0001) compared to those with a single healthcare provider, and those with no healthcare provider had a significantly lower odds of CHD (OR, 0.32; 95% CI, 0.27-0.38; *P *< 0.0001) (Figure 3C).

**Table 2:** Health status and healthcare access of the study population. CHIP, Children's Health Insurance Program; VA, Veterans Affairs; CHAMP-VA, Civilian Health and Medical Program of the Department of Veterans Affairs

Variable	*n* (%)
Health status	
Mental health (*n* = 22,353)	
0 days when mental health is not good (REF)	14,277 (60.5)
1-13 days when mental health is not good	4,413 (20)
14+ days when mental health is not good	3,663 (19.5)
Physical health (*n* = 22,033)	
0 days when physical health is not good (REF)	10,197 (45.2)
1-13 days when physical health is not good	5,089 (23)
14+ days when physical health is not good	6,747 (31.7)
Healthcare provider (*n* = 22,753)	
Yes, only one (REF)	11,032 (46.8)
More than one	10,969 (48.7)
No	752 (4.5)
Insurance (*n* = 22,182)	
A plan purchased through employer/union (REF)	3,431 (19.1)
A private plan bought on your own	1,323 (7.6)
Medicare	13,541 (53.5)
Medigap	32 (0.2)
Medicaid	1,354 (7.2)
Children's Health Insurance Program (CHIP)	1
Military-related healthcare (TRICARE/VA/CHAMP-VA)	1,059 (4)
Indian Health Service	59 (0.1)
State-sponsored health plan	432 (2.6)
Other government programs	534 (2.6)
No coverage of any type	416 (3.1)
Past 12 months, needed to see a doctor but could not afford it (MEDCOST1) (*n* = 22,813)	
Yes (REF)	1,672 (9.7)
No	21,141 (90.3)

Healthcare access

**Figure 4:**
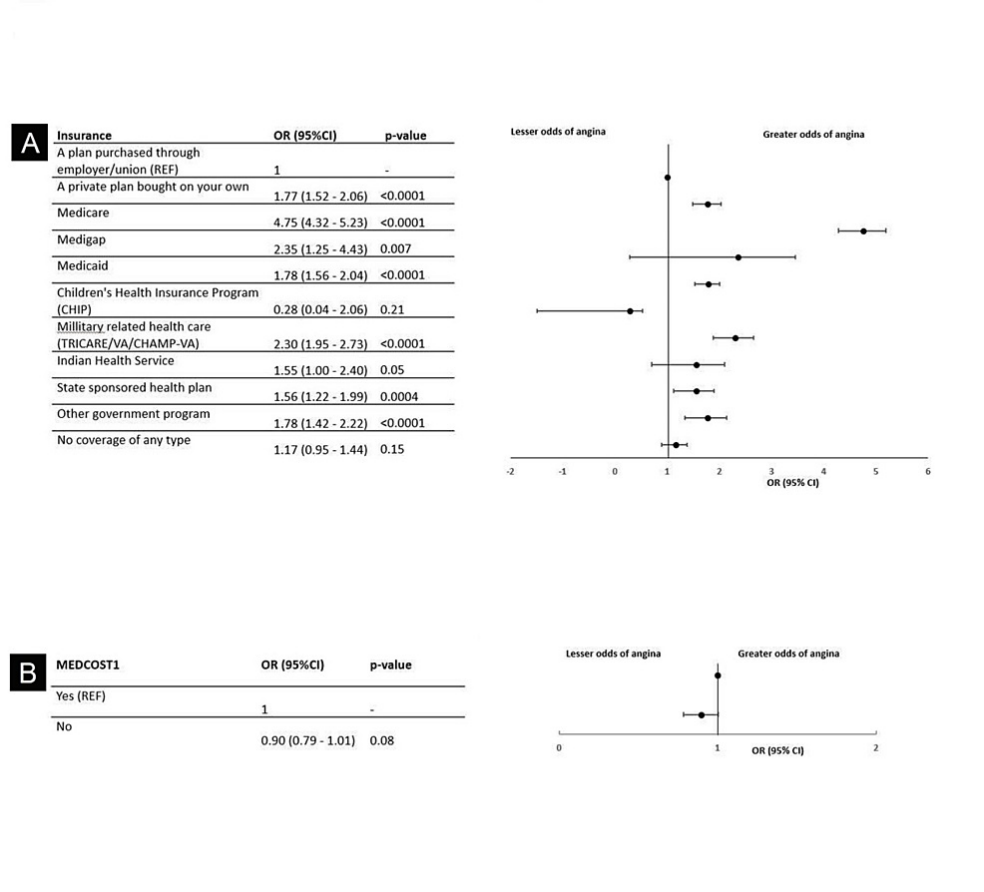
Odds of CHD stratified by health status and healthcare access: (A) insurance types and (B) costs. CHD, coronary heart disease

When considering insurance options among the 22,182 respondents, those with Medicare had the highest odds of CHD (OR, 6.30; 95% CI, 5.75-6.90; *P *< 0.0001) compared to respondents with a plan purchased through an employer or union as the reference group (Figure 4A). No significant association has been found between the ability to afford medical care and odds of having CHD (*P *> 0.05) (Figure 4B).

## Discussion

The study aimed to investigate the prevalence disparities of CHD across demographic, socioeconomic, and health factors in the U.S. population using BRFSS data. The key findings revealed significant associations between CHD prevalence and various factors. Among the noteworthy results, individuals aged 65 and above exhibited higher odds of CHD, while females demonstrated lower risk compared to males. Hispanics, among all races, presented the lowest odds of CHD. Socioeconomic factors such as unemployment, retirees, and income below $20,000 but >$15,000 were associated with higher CHD odds. Poor mental and physical health, multiple healthcare providers, and Medicare insurance users also showed increased CHD odds.

CHD is considered the most prevalent form of heart disease and is a cause of major morbidity and mortality in the United States [11]. The results of this study demonstrated associations between CHD, defined as a condition in which there is an inadequate supply of blood and oxygen to the myocardium due to atheromatous changes, and other parameters like socioeconomic, demographic, health status, and healthcare access [12].

The study revealed that among the respondents with a history of CHD, 59% were 65 years old or older. A 2017 study by Jaul and Barron, concluded that the percentage of the national population over age 65 has increased in the last 10 years and will continue to rise for another 20 years due to improved life expectancies and a post-war baby boom. Starting in 2030, the number of adults over age 85 will rise quickly [13]. This finding is alarming considering that this study revealed that compared to the reference age group of 18 to 24 years, the odds of developing CHD in the 65+ age group were significantly higher. According to a study by Prince et al., the leading contributor to the burden of disease in elderly patients is related diseases, accounting for over 30% of the total burden in older people. As this population continues to age and more respondents move into this age group, primary prevention must be implemented in populations under 65 years of age to ensure that manageable risk factors are attended to [14].

This study observed a significantly higher risk among respondents who have an annual household income range of $15,000 to $20,000 to develop CHD. Comparatively, there was no significant relationship between income and history of CHD among respondents with income over $50,000 compared to the reference group. A study conducted by Stronks et al. also reported similar observations, among those in the lowest income group, the risk of bad perceived health was three times as high in contrast to people in the highest income group [15]. We also found that among 22,593 respondents, individuals who were unable to work or unemployed for longer periods and retirees had significantly higher odds of CHD. A study by Herbig et al. concluded that the long-term unemployed carry a significantly higher burden of disease than employed individuals and those who are unemployed only for a short time. The burden of disease increases with the duration of unemployment [16]. The cycle of unemployment lower household income and higher risk of CHD can be managed by attending to controllable risk factors and having community and additional support programs set up to promote and support low-income, unemployed and retired individuals.

Of the 22,753 respondents involved in the study, 48.7% of individuals with more than one healthcare provider had significantly higher odds of CHD compared to those with a single healthcare provider. Concurrently, those with no healthcare provider had significantly lower odds of CHD. When patients are diagnosed and manage multiple diagnoses, it is common to seek multiple healthcare providers. Comorbidities often require multiple healthcare providers to help manage these chronic conditions. Individuals within this category also often have multiple risk factors and increased stress with the management of multiple diagnoses. in a study by Rayman et al., people with multimorbidity have poorer functional status, quality of life, and health outcomes, and are higher users of ambulatory and inpatient care than those without multimorbidity [17]. These factors often collectively increase the odds of developing even more diseases such as CHD. In contrast, if an individual only seeks care from a healthcare provider for 1 diagnosis or doesn't have a need to seek help at all, the individual is typically in a better state of health and limits some risk factors that individuals with multimorbidities would not be able to avoid.

When considering insurance options among the 22,182 respondents, respondents with Medicare had the highest odds of CHD compared to the reference group of respondents with a plan purchased through an employer or union. Patients often consider their means of affording or compensating the health care institution for services and seeking medical treatment and advice. In a study by Riley, the conclusion was made that the consequences of being uninsured are significant and include the use of fewer preventive services, poorer health outcomes, higher mortality and disability rates, lower annual earnings because of sickness and disease, and the advanced stage of illness (i.e., many are *sicker* when diagnosed) [18]. It is not uncommon for an individual to be cautious about seeking care due to the lack of insurance or poor coverage. These individuals also face several limitations on the road to recovery due to the lack of preventative measures taken.

The data from this study may form the framework for a discussion on the physician’s role in addressing healthcare access disparities. There are clear correlations between developing CHD and being in the age population of 65+, having an annual household income range of $15,000 to $20,000, having more than one healthcare provider, or lower access to healthcare. Patients are often hesitant to seek care until their condition is critical due to a variety of factors such as costs and lack of knowledge about their health. Increasing public awareness of these disparities is critical to furthering research on the subject and fueling targeted healthy policy initiatives. At a local level, doctors and healthcare organizations must devise considerable options to close the access gap such as opening up telemedicine opportunities for patients who are located remotely and unable to travel to the hospital or clinic. Doctors and healthcare organizations should also consider creating and promoting free reliable resources for individuals to use at home to help supply information and supportive measures. In multiple studies such as the ones conducted by Nyshita et al. and Mylavarapu et al., it was observed that although YouTube videos uploaded by doctors and hospitals had less reach among viewers, the videos were observed to be of good quality and very reliable [19,20]. Organizations and healthcare providers should ensure that patients have access to accurate and reliable information, at reduced or free cost which is vital in their health decision-making [19,20]. Together with the help of state advocacy groups, healthcare providers and policy makers, it would be beneficial to identify gaps and causes of disparities and then take a collective approach to benefit individuals and implement policy change.

Limitations

It is pertinent to acknowledge limitations concerning the study. Using the data provided by BRFSS comes with its own shortcomings. Because the survey was conducted via telephone, a large proportion of the population did not respond, resulting in a nonresponse bias. Nonresponse bias could lead to a sample that was not representative of the whole population, affecting the statistical parameters, such as prevalence. Moreover, the study utilized the 2021 BRFSS dataset, which might not reflect the most current trends and conditions, and relied on self-reporting, which might result in recall bias, social desirability bias, and response bias, potentially affecting the accuracy of the data. Bias might also have resulted from our exclusion criteria, as a few few race and ethnicity combinations were excluded. This exclusion could have limited the external validity of the study findings. The retrospective nature of the study design made it difficult to control all potential confounding variables, leading to confounding bias, which can create spurious associations between nondependent variables and dependent variables. We could not establish the causation of the disease. Nonresponse bias could have been reduced by making multiple contacts and making the process of conducting surveys user-friendly. Problems with self-reporting could have been reduced by using neutral language to avoid bias from leading questions, assuring the participants that the survey will be confidential, and providing memory aids to recall more effectively.

## Conclusions

Older adults, particularly those aged ≥65 years, face a higher risk of developing CHD as per our study. Socioeconomic factors, such as lower income and unemployment, significantly impact CHD risk. Community support programs are crucial to mitigate these risk factors. Poor health status and multiple healthcare providers are identified as critical determinants of CHD risk, emphasizing the need for integrated care models. Access to healthcare, especially insurance coverage, influences CHD risk, with Medicare recipients having the highest odds.

A comprehensive plan is, therefore, essential to address CHD disparities, involving public health measures, community support, and policy reforms. Public awareness, preventive measures, and improved healthcare access, including telemedicine, are crucial. Efforts to mitigate the study limitations could include making multiple contact attempts to reduce nonresponse bias, using neutral language to minimize bias in self-reporting, ensuring confidentiality to encourage honest responses, and employing memory aids to improve recall accuracy. Despite the limitations, the study provides valuable insights for future research and policy development to combat CHD, a leading cause of disease and death in the United States.

## References

[1] Kloner RA, Chaitman B (2017). Angina and its management. J Cardiovasc Pharmacol Ther.

[2] Duggan JP, Peters AS, Trachiotis GD, Antevil JL (2022). Epidemiology of coronary artery disease. Surg Clin North Am.

[3] Youmans QR, Hastings-Spaine L, Princewill O, Shobayo T, Okwuosa IS (2019). Disparities in cardiovascular care: past, present, and solutions. Cleve Clin J Med.

[4] Niakouei A, Tehrani M, Fulton L (2020). Health disparities and cardiovascular disease. Healthcare (Basel).

[5] Bansal A, Hiwale K (2023). Updates in the management of coronary artery disease: a review article. Cureus.

[6] McPherson R, Tybjaerg-Hansen A (2016). Genetics of coronary artery disease. Circ Res.

[7] Bauersachs R, Zeymer U, Brière JB, Marre C, Bowrin K, Huelsebeck M (2019). Burden of coronary artery disease and peripheral artery disease: a literature review. Cardiovasc Ther.

[8] (2021). Centers for Disease Control and Prevention (CDC). Behavioral Risk Factor Surveillance System Survey Data. https://stacks.cdc.gov/view/cdc/21784/cdc_21784_DS1.pdf.

[9] Rolle-Lake L, Robbins E (2024). Behavioral Risk Factor Surveillance System. https://www.ncbi.nlm.nih.gov/books/NBK553031/.

[10] Jacobsen S, Ladd C, Streck S (2021). Research Committee Approval for Use of the Behavioral Risk Factor Surveillance System Data: A Cross-Sectional Study. Oklahoma State University Center for Health Sciences Research Days 2021.

[11] Shahjehan RD, Bhutta BS (2024). Coronary Artery Disease. https://www.ncbi.nlm.nih.gov/books/NBK564304/.

[12] Regmi M, Siccardi MA (2024). Coronary Artery Disease Prevention. https://www.ncbi.nlm.nih.gov/books/NBK547760/.

[13] Jaul E, Barron J (2017). Age-related diseases and clinical and public health implications for the 85 years old and over population. Front Public Health.

[14] Prince MJ, Wu F, Guo Y (2015). The burden of disease in older people and implications for health policy and practice. Lancet.

[15] Stronks K, van de Mheen HD, Mackenbach JP (1998). A higher prevalence of health problems in low income groups: does it reflect relative deprivation?. J Epidemiol Community Health.

[16] Herbig B, Dragano N, Angerer P (2013). Health in the long-term unemployed. Dtsch Arztebl Int.

[17] Rayman G, Akpan A, Cowie M, Evans R, Patel M, Posporelis S, Walsh K (2022). Managing patients with comorbidities: future models of care. Future Healthc J.

[18] Riley WJ (2012). Health disparities: gaps in access, quality and affordability of medical care. Trans Am Clin Climatol Assoc.

[19] Naga Nyshita V, Kuruvila M, Galidevara S, Sundaram A, Sirohi S, Singh M (2023). YouTube as a patient information source for gastrointestinal reflux disease. Cureus.

[20] Mylavarapu M, Maheta D, Clarke S, Parmar K, Mohammed M, Vuyyuru CS (2023). Diabetes mellitus on YouTube: a cross-sectional observational study to assess the quality and reliability of videos. Cureus.

